# Association between Periodontitis and Genetic Polymorphisms in Interleukins among Patients with Diabetes Mellitus

**DOI:** 10.3390/dj9040045

**Published:** 2021-04-18

**Authors:** Hoda M. Abdellatif, Munerah Saleh Binshabaib, Heba A. Shawky, Shatha Subhi ALHarthi

**Affiliations:** Preventive Dental Sciences Department Periodontology Division, College of Dentistry, Princess Nourah Bint Abdulrahman University, Riyadh P.O. Box 84428, Saudi Arabia; hodamaabdellatif@gmail.com (H.M.A.); MSBinShabaib@pnu.edu.sa (M.S.B.); haelsabagh@pnu.edu.sa (H.A.S.)

**Keywords:** interleukin, genetic polymorphisms, periodontitis, diabetes mellitus

## Abstract

There is a perplexity in the association between interleukin (IL) polymorphisms and periodontitis among patients with and without diabetes mellitus (DM). The aim of the present study was to evaluate indexed data regarding the association between periodontitis and genetic polymorphisms in interleukins among patients with and without DM. The addressed question was “Is there an association between periodontitis and polymorphisms in interleukins among patients with and without DM?” Original studies were included. Indexed databases were searched, and the pattern of the present literature review was customized to summaries’ the pertinent information. Eight studies were included and processed for data extraction. Two studies showed that polymorphisms in IL-1B genes aggravate periodontitis in patients with type-2 DM, and two studies showed that IL-1B genes either do not or are less likely to contribute towards the progression of periodontitis in patients with type-2 DM. Two studies reported that IL genes do not show cross-susceptibility with periodontitis and type-2 DM. One study reported that the primary factor that governs the occurrence and progression of periodontitis in patients with and without type-2 DM is poor routine oral hygiene maintenance. Seven studies had a high risk of bias. The role of IL gene polymorphisms in the development and progression of periodontitis in patients with and without DM remains controversial.

## 1. Introduction

The bidirectional relationship between periodontitis and diabetes mellitus (DM) is well-reported [1,2]. Studies [3,4,5,6] have shown that chronic hyperglycemia, which is an evident manifestation in patients with poorly controlled DM and prediabetes, is a risk factor of periodontal soft tissue inflammation and marginal bone loss (MBL). From the biomechanics perspective, a state of persistent hyperglycemia has long been criticized for aggravating periodontal disease clinical signs such as clinical attachment loss, increased probing depth, and MBL because it accelerates the formation and accumulation of advanced glycation end-products (AGESs) in periodontal tissues [7,8,9]. Interactions between AGEs and their receptors (RAGEs) induce a state of oxidative stress in periodontal tissues [2] that, in turn, augments the production of destructive inflammatory cytokines such as interleukin (IL) 1 beta (1β), IL-6, and tumor necrosis factor alpha (TNF-α) [4,10]. Furthermore, raised blood glucose levels activate the nuclear factor kappa-light-chain-enhancer of activated B cells (NF-κB), which induce changes in gene expression in periodontal fibroblasts and accelerate MBL [11].

In a systematic review and meta-analysis, Zao and Ronghua [12] investigated the connotation between IL-6 polymorphism and periodontitis. The results demonstrated that IL-6 polymorphisms negatively affect periodontitis [12]. On the contrary, in another review, Yin et al. [13] suggested that results of studies that assessed the relationship between IL-6 polymorphisms and periodontitis should be interpreted with deep caution due to the high level of heterogeneity among the included studies. This clearly reflects that there is a disagreement in relation to the association between IL-6 polymorphisms and periodontitis. Furthermore, with reference to periodontitis and DM, studies with conflicting results have investigated variations in IL gene clusters [14,15,16,17,18,19]. In a pilot investigation, Kavitha et al. [16] assessed the association between single nucleotide polymorphisms in the IL-6 gene with periodontitis and type-2 DM. The results showed that the IL-6 genotype is a high-risk gene associated with the development of periodontitis in patients with and without type-2 DM [16]. Similar results were reported by Xiao et al. [20], Raunio et al. [21], and Struch et al. [22] In an in-vitro study, Shi et al. [23] reported that IL-10 modulates the local host response by reducing periodontal inflammation. However, controversial results have also been reported in this context. López et al. [15] investigated the association between periodontitis, type-2 DM, and IL-1 gene polymorphisms. This association was assessed among 112 type-2 DM patients with periodontitis, 224 non-diabetic patients with periodontitis, and 208 systemically healthy individuals without periodontitis. The results showed that polymorphisms were significantly associated with IL-1 gene in patients with periodontitis [15]; however, there was no significant relationship between type-2 DM and Interleukin 1 Alpha (IL-1A) and Interleukin 1 Beta (IL-1B) gene polymorphisms [15]. Similarly, in the studies by da Silva et al. [24] and Ding et al. [25], no significant association between IL-1A and Interleukin 1 Receptor Antagonist (IL-1RN) variants among patients with and without periodontitis was found. However, it is noteworthy that in these studies [24,25], patients with DM were not sought. Another study that has confused the role of gene polymorphisms in the progression of periodontitis in patients with and without DM was the one by Deppe et al. [17], in which the authors showed that poor oral hygiene is the main factor that governs the progression of periodontitis in vulnerable populations. According to Deppe et al. [17], the IL genotype plays an insignificant role in the progression of periodontitis in patients with and without type-2 DM, whereas experimental results by Toker et al. [26] showed that IL-10 gene polymorphism is associated with an increased susceptibility to periodontitis. In this context, there is a perplexity in the association between IL polymorphisms and periodontitis among patients with and without type-2 DM. The authors of the present study hypothesized that there is no association between genetic polymorphisms and periodontitis in patients with DM.

The aim of the present study was to evaluate indexed data regarding the association between periodontitis and IL polymorphisms among patients with and without type-2 DM.

## 2. Materials and Methods

### 2.1. Focused Question

The question under consideration was “Is there an association between periodontitis and polymorphisms in interleukins among patients with and without type-2 DM?”

### 2.2. Eligibility Criteria and Literature Search

Only original clinical and experimental studies were eligible for inclusion. In other words, case-series, commentaries, letters to the editor, case-reports, and perspectives were not sought.

**Figure 1:**
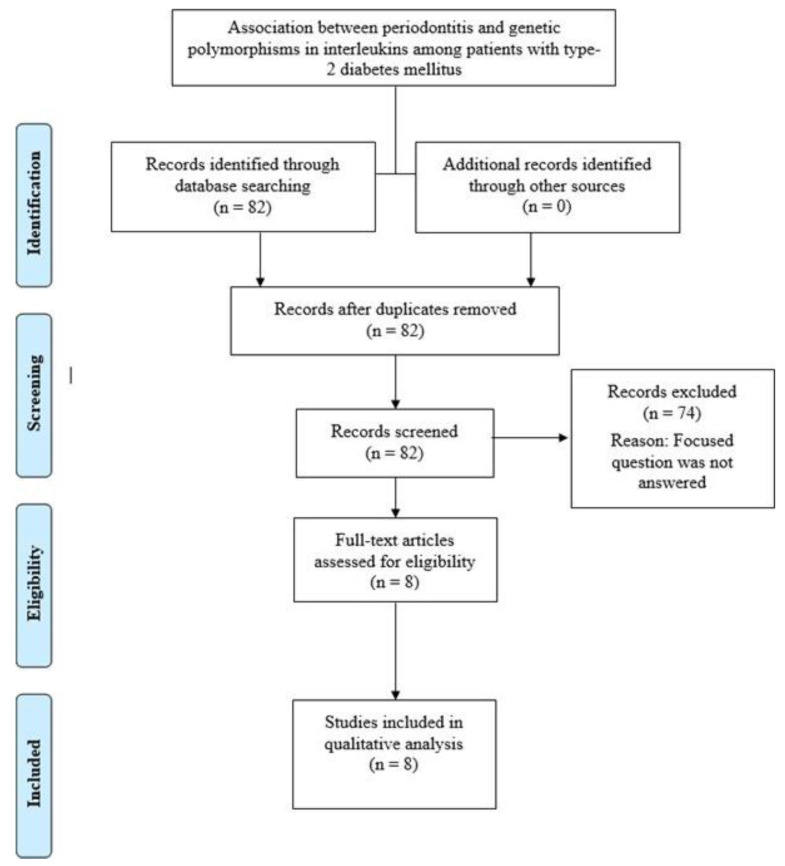
Preferred Reporting Items of Systematic Reviews and Meta-Analysis (PRISMA) flow diagram for the research protocol.

In order to identify the pertinent studies, an electronic search without time and/or language restrictions was conducted up to and including December 2020 using the PubMed, Scpous, EmbasE, Google Scholar, and Web of Knowledge databases. Different combinations of the following medical subject headings (MeSH) were used: alveolar bone loss, cytokine, type-2 diabetes mellitus, inflammation, interleukin, and periodontitis. Boolean operators (OR and AND) were used to combine the keywords to expand the search outcomes. The titles and abstracts of studies identified using the above-described protocol were screened by two authors and checked for agreement. The authors independently comprehended the full-texts of relevant studies. In addition, the reference lists of pertinent studies were hand-searched. Disagreements related to articles selection were settled by discussion. The literature search was performed in accordance with the Preferred Reporting Items of Systematic Reviews and Meta-Analysis (PRISMA) guidelines, as shown in Figure 1.

The scale by Downs and Black [27] was utilized for the assessment of risk of bias (RoB). Scores of studies of ≥20, 15–19, and <14 were considered good, fair, and poor, respectively [27]. One author assessed the RoB across the studies.

## 3. Results

### 3.1. General Characteristics

Eighty-two articles were initially identified, out of which 74 articles that did not fulfill the eligibility criteria were excluded. Eight studies [14,15,16,17,18,19,20,22] were included and processed for data extraction. In all studies [14,15,16,17,18,19,20,22], the diagnosis of periodontitis was based on the presence of periodontal pockets of probing depths of at least 4 mm and clinical attachment loss of at least 3 mm in 30% of sites. Seven studies [14,15,17,18,19,20,22] had a cross-sectional case-control design, and one study [16] was a pilot investigation. Seven studies [15,16,17,18,19,20,22] were performed in type-2 diabetic and non-diabetic individuals with and without periodontitis. In one study [14], type-1 and type-2 diabetic patients and systemically healthy individuals with and without periodontitis were included. In three [15,18,22] and four studies [14,15,18,22], IL-1A and IL-1B gene polymorphisms, respectively, were assessed. In one study [18], IL-1A, IL-1B, IL-4, IL-6, and IL-10 gene polymorphisms were assessed. In all studies, IL polymorphisms were assessed using polymerase chain reaction (Table 1).

### 3.2. Outcomes

Results from two studies [14,22] showed that polymorphisms in IL-1B genes aggravates periodontitis in patients with type-2 DM, and outcomes from two studies [15,18] showed that IL-1B genes either do not or are less likely to contribute to the progression of periodontitis in patients with type-2 DM. Results by Deppe et al. [17] and Petrovic et al. [19] showed that IL genes do not show cross-susceptibility with periodontitis and type-2 DM. In a study by López et al. [15], the IL-1A genotype was not associated with the progression of periodontitis in patients with type-2 DM, and Struch et al. [22] reported that the IL-1A genotype aggravates periodontitis in patients with type-2 DM. Two studies reported that the IL-6 allele is a protective factor of periodontitis and DM progression. According to Deppe et al. [17], the primary factor that governs the occurrence and progression of periodontitis in patients with and without type-2 DM is poor routine oral hygiene maintenance, and the role of IL gene polymorphisms in this regard is insignificant [17]. These results are shown in Table 1.

### 3.3. Risk of Bias Assessment

Seven [14,15,16,18,19,20,22] of the eight [14,15,16,17,18,19,20,22] included studies had a high risk of bias. The study by Deppe et al. [17] had a low risk of bias (Table 2).

## 4. Discussion

Based upon the focused question, the objective of the present study was to review indexed literature that assessed the role of IL gene polymorphisms in the progression and of periodontitis among patients with and without type-2 DM. The authors endeavored to perform a systematic review and meta-analysis of the studies that addressed the focused question; however, a number of methodological limitations of the included studies complicated the quantitate and qualitative assessment of the data derived from the assessed studies. It is well-established that the definition of patients, intervention/exposure, control, and outcome (PICO or PECO) comprise fundamental criteria for systematic reviews [28,29]. It is noteworthy that the PECO parameters varied among the assessed studies. For instance, in the studies by Deppe et al. [17] and Struch et al. [22], patients were divided into two groups, i.e., individuals with and without type-2 DM; whereas in studies by Borilova Linhartova et al. [14], López et al. [15], and Kavitha et al. [16], patients were divided into multiple groups that encompassed systemically healthy and type-2 diabetic patients with and without periodontitis. In this context, it was demanding to precisely define the patients and control groups for a standardized PICO/PECO protocol.

With reference to the obtained results, it is difficult to reach a definitive consensus regarding the relationship between IL gene polymorphisms and periodontitis among patients with and without type-2 DM. For instance, according to results reported in the study by Borilova Linhartova et al. [14] the IL-1B∗T allele is protective against the occurrence of periodontitis; however, these results contradicted those reported by da Silva et al. [24], which associated T allele in Caucasian carriers with a 1.25-times higher risk of developing periodontitis than C allele carriers. Nevertheless, from the authors’ perspective, IL gene polymorphisms cannot be solely held liable to the progression and periodontitis in patients with and without DM. The authors applaud the study by Deppe et al. [17], according to which, the progression of periodontitis in patients with type-2 DM is significantly associated with impaired routine oral hygiene maintenance. Studies [30,31,32,33] have shown that the unstimulated whole salivary flow rate is significantly low than its normal threshold (0.2 mL/min) in patients with poorly-controlled DM and prediabetes. This leads to an increased accumulation of the oral biofilm in supra- and sub-gingival teeth surfaces, thereby exposing periodontal soft and osseous tissues to periodontopathogenic microbes including *Aggregatibacter actinomycetemcomitans*, *Porphyromonas gingivalis*, *Prevotella intermedia*, *Treponema denticola,* and *Enterococcus* species [34]. The role of persistent hyperglycemia, which is manifested in patients with poorly-controlled type-1 and type-2 DM and prediabetes, cannot be disregarded [3,5,10,35]. Moreover, chronic hyperglycemia induces oxidative stress in tissues, which dysregulates bone turnover [36]. Simultaneously, an increased production of AGEs in hyperglycemic patients further compromises osseous remodeling and delays healing [37,38,39,40]. Nevertheless, it has also been documented that under optimal glycemic control, patients with DM can have a healthy periodontal status, which is similar to that observed in systemically-healthy individuals without periodontitis [6,33,41]. This suggests that poor oral hygiene and the presence of periodontopathogenic bacteria play major roles in the progression of periodontitis in patients with and without DM, as well as that the role of gene polymorphisms in this regard is rather secondary. Though the contribution of IL gene polymorphisms remains controversial, it has been shown that under optimal glycemic control, genetic polymorphisms are markedly reduced in patients with DM [6]. Further well-designed and power-adjusted studies are needed to assess the aforementioned hypotheses.

It is important to mention that nearly 88% of studies had a high risk of bias. Furthermore, a prior sample-size estimation was performed only in one [17] of the eight assessed studies [14,15,16,17,18,19,20,22]. In this context, the results of the assessed studies [14,15,16,17,18,19,20,22] should be interpreted with deep caution.

## 5. Conclusions

The role of IL gene polymorphisms in the development and progression of periodontitis in patients with and without DM remains controversial.

## Figures and Tables

**Table 1:** Characteristics of the studies included.

Author et al.	Study Design	Patients (n)	Age Range	Groups	Assessed Interleukin Polymorphisms	Outcome
Borilova Linhartova et al. [14]	CS/CC	1106 patients	30–77 years	Gp-1: Patients with periodontitis Gp-2: Patients with type-1 DM Gp-3: Patients with periodontitis and type-1 DM Gp-4: Patients with type-2 DM Gp-5: Patients with periodontitis and type-2 DM	IL-1 gene	IL-1B polymorphism is associated with periodontitis. IL-1RN variant is associated with T1DM
López et al. [15]	CS/CC	544 patients	42–68 years	Gp-1: Patients with periodontitis Gp-2: Patients with type-2 DM Gp-3: Patients without type-2 DM and periodontitis	IL-1 gene	IL-1A/1B genotype is not associated with type-2 DM. IL-1 genotype is a risk allele for the development of periodontitis.
Kavitha et al. [16]	CC	120 patients	NR	Gp-1: Patients with periodontitis Gp-2: Patients with type-2 DM Gp-3: Patients with periodontitis and type-2 DM Gp-4: Patients without type-2 DM and periodontitis	IL-6 gene	IL-6 genotype is a risk allele for the development of periodontitis. IL-1 genotype is not associated with type-2 DM.
Deppe et al. [17]	CC	104 patients	48–72 years	Gp-1: Patients with type-2 DM Gp-2: Patients without type-2 DM	IL-1 gene	IL genotypes do not play a role in the progression of periodontitis Poor oral hygiene is a more reliable predictor of periodontitis in patients with and without type-2 DM
Cirelli et al. [18]	CS/CC	894 patients		Gp-1: Patients with periodontitis Gp-2: Patients with periodontitis Gp-3: Patients with type-2 DM	IL-1A, IL-1B, IL-4, IL-6 and IL-10	IL-1B genotype is less likely associated with periodontitis and type-2 DM. IL-4 and IL-6 genotypes are more likely associated with periodontitis and type-2 DM.
Petrovic et al. [19]	CC			Gp-1: Patients with periodontitis Gp-2: Patients with periodontitis and type-2 DM Gp-3: Patients without type-2 DM and periodontitis		IL genes did not show cross-susceptibility with periodontitis and type-2 DM
Xiao et al. [20]	CS/CC	492 patients	40–87 years	Gp-1: Patients with periodontitis Gp-2: Patients with type-2 DM Gp-3: Patients with periodontitis and type-2 DM Gp-4: Patients without type-2 DM and periodontitis	IL-6 gene	Patients in Gp-3 showed the lowest IL-6 genotype and C allele frequencies compared with other groups. The IL-6-572 C allele is a protective factor of diseases progression.
Struch et al. [22]	CS/CC	1515 patients	40–60 years	Gp-1: Patients with type-2 DM Gp-2: Patients without type-2 DM	IL-1A/1B haplotype	IL-1A/1B genotype aggravates periodontitis in patients with type-2 DM.

CS: cross-sectional; CC: case-control; NR: not reported; DM: diabetes mellitus; IL: interleukin.

**Table 2:** Risk of bias assessment using the Downs and Black [27] tool.

Author et al.	Reporting (Range: 2–12)	External Validity (Range: 0–2)	Bias (Range: 0–6)	Confounding (Range: 1–6)	Power (Range: 0–1)	Total	Final Assessment
Borilova Linhartova et al. [14]	4	1	0	2	0	7	High
López et al. [15]	6	1	2	3	0	12	High
Kavitha et al. [16]	4	1	2	2	0	9	High
Deppe et al. [17]	8	4	4	5	1	22	Low
Cirelli et al. [18]	5	1	1	0	0	7	High
Petrovic et al. [19]	4	1	2	2	0	9	High
Xiao et al. [20]	5	1	1	2	0	9	High
Struch et al. [22]	3	1	1	2	0	7	High

## Data Availability

Data is available on reasonable request.
